# How Age and Disclosures of Sponsored Influencer Videos Affect Adolescents’ Knowledge of Persuasion and Persuasion

**DOI:** 10.1007/s10964-019-01191-z

**Published:** 2020-01-18

**Authors:** Eva A. van Reijmersdal, Sophia van Dam

**Affiliations:** 1 Amsterdam School of Communication Research ASCoR, PO Box 15791, 1001 NG Amsterdam, The Netherlands; 2 Dutch Institute for Nature Education (IVN), Amsterdam, The Netherlands

**Keywords:** Disclosure, Adolescents, Persuasion knowledge, Influencer marketing, Advertising, Product placement

## Abstract

This study examines the effects of age (early versus middle adolescence) combined with the content of disclosures for sponsoring in online influencer videos on adolescents’ knowledge of persuasion and on persuasion. An experiment was conducted among a sample of 406 adolescents (167 early adolescents aged 12–14 years, mean age 12.85, *SD* = 0.14, 53% female; and 239 middle adolescents, aged 15–16 years, mean age 14.36, *SD* = 0.13, 59% female). The results show that early adolescents need extensive information (disclosure of advertising and of its intent) to activate their knowledge of persuasion regarding sponsored influencer videos, whereas middle adolescents’ knowledge of persuasion is activated by disclosure of advertising alone. This indicates that early adolescents’ knowledge of persuasion is less well developed and that their information processing is more limited than that of middle adolescents. Moreover, only middle adolescents showed more negative brand and influencer attitudes in response to the disclosure. Interestingly, purchase intention remains unaffected by the disclosure for all adolescents. These findings add to the research on adolescence as they show that adolescents’ responses to influencer marketing are a function of their developmental stage in combination with disclosure content. As such, this study has implications for theory on persuasion among adolescents and for regulations aiming to empower adolescents to deal with online sponsored influencer videos.

## Introduction

Currently, online influencer marketing is a popular way for brands to reach young consumers. With influencer marketing, brands pay influencers to mention or use the brand in their content. Online influencers are “people who built a network of followers and are regarded as trusted tastemakers in one or several niches” (De Veirman et al. 2017, p. 1). The ethics of this integrated and hidden way of advertising are extensively discussed because the persuasive message is difficult to recognize. Disclosures of influencer marketing are proposed as a remedy for a more transparent online media environment that can reduce unwitting persuasion. However, insights into whether these labels (or disclosures) help young consumers to recognize influencer marketing as a form of advertising are limited. Previous research on disclosures has mostly focused on adults or younger children, assuming that from the age 12, children’s knowledge of persuasion has reached adult levels (for an overview see, Boerman and van Reijmersdal 2016). However, several studies have shown that adolescents should be treated differently than children and adults (Livingstone and Helsper 2006; Van Reijmersdal et al. 2017), as adolescents seem susceptible to influencer advertising due to their underdeveloped knowledge of persuasion combined with adolescents’ limited cognitive skills, hyper-responsive emotional system, and socio-emotional development (Pechmann et al. 2005).

Moreover, persuasion knowledge levels are expected to vary between different stages of adolescence (Livingstone and Helsper 2006). For example, understanding of advertising’s intentions asks for cognitive abilities that are developed at an older age than being able to easily recognize advertising (John 1999). Consequently, early adolescents might need more extensive disclosures (including information about advertising’s intentions) than middle adolescents to help them active their knowledge of persuasion. To test these assumptions, this study aims to investigate the effects of two disclosure types (i.e., disclosure of advertising and disclosure of advertising and its intentions) on the activation of knowledge of persuasion and on persuasion. Furthermore, this study investigates the role of different stages of adolescence (i.e., early adolescence vs middle adolescence) in disclosure effects.

### Effects of Disclosure Type on Conceptual Knowledge of Persuasion

The goal of disclosures is to inform consumers and trigger their knowledge of persuasion. If consumers, and adolescents in particular, are unaware of the persuasive nature of influencer marketing, they cannot defend themselves against the persuasion attempt to the same extent as when they knew they were being persuaded (Hudders et al. 2017). This makes adolescents more susceptible to persuasion, which poses serious threats to adolescents’ individual well-being, especially when harmful products are promoted, such as alcohol or unhealthy foods.

Activation of knowledge of persuasion entails the activation of its two dimensions: conceptual knowledge of persuasion and attitudinal knowledge of persuasion (Rozendaal et al. 2011). Conceptual knowledge of persuasion consists of several elements of which recognition of sponsored content as being advertising and understanding advertising’s intentions are seen as the basics (Rozendaal et al. 2011). Recognition of sponsored content as being advertising refers to the ability to recognize certain content as being advertising and the fact that the advertiser is the source of the message (Wojdynski and Evans 2019). It is unlikely that one will reflect on advertising when it is not recognized as such. Therefore, recognition of sponsored content as being advertising is a fundamental step in the activation of knowledge of persuasion. This could be achieved by adding, for example, a third-party disclosure to sponsored influencer content, which states that the influencer advertises a certain brand within that specific content (Wojdynski and Evans 2019). By disclosing this information, adolescents are alerted to the fact that the influencer content contains advertising. By showing such a disclosure, a priming effect may occur: among adults, research showed that as the disclosed information (e.g., that the content contains advertising) is fresh in the viewer’s memory, it is easy to access while processing the content (Van Reijmersdal et al. 2013). Subsequently, commercial content will be more easily recognized. A disclosure that reports that the content includes advertising messages is assumed to help adolescents to improve their recognition that the content is a form of advertising (Wojdynski and Evans 2016).

The second aspect of conceptual knowledge of persuasion is the understanding that advertising has the intent to persuade and to sell (Robertson and Rossiter 1974). This is more complex than solely recognizing a commercial message and might require more cognitive capacity. Children may discriminate between advertisements and programs based on characteristics of the content (that is, their recognition of advertising is activated), without understanding the intent of advertisements, namely that they try to persuade (Gunter et al. 2005). Based on research among adults, it is expected that it might be effective to disclose not only that the sponsored content contains advertising but also the actual goal of the sponsored content (i.e., to persuade), to improve adolescents’ understanding of advertising intentions (Van Reijmersdal 2016).

### Moderating Effect of Age on Conceptual Knowledge of Persuasion

While the embedded nature of online influencer marketing limits the activation of knowledge of persuasion, there is also a question about whether adolescents’ knowledge of persuasion has already reached mature levels (Boush et al. 1994). Although scholars agree that children do not possess the same cognitive capacity for understanding advertising as adults (Rozendaal et al. 2010), there is disagreement on how much children do know and understand and at what age this knowledge and understanding fully develop (Lapierre and Rozendaal 2019). On the one hand, some studies indicated that children from 12 years old do possess a critical understanding of advertising and its intentions (Boush et al. 1994). On the other hand, some authors question the belief that adolescents are as knowledgeable and critical as adults (Livingstone and Helsper 2006; Rozendaal et al. 2011). In particular, studies indicate the limited knowledge of both children and adolescents (7 to 14 years old) with regard to integrated advertising techniques (Verhellen et al. 2014). Lawlor et al. (2016) studied knowledge of persuasion among early adolescents (12 to 14 years old) and indicated that these children still had difficulty recognizing commercial messages in online social network sites.

Next to their limited general knowledge of persuasion, adolescence is a very specific period in life marked by cognitive and social-emotional development (Valkenburg and Piotrowski 2017), which decreases adolescents’ ability to activate knowledge of persuasion. Adolescence can be divided into three stages: early adolescence, middle adolescence, and late adolescence (Spano 2004), each with its own characteristics. The current study will make a distinction between teenagers in early adolescence and middle adolescence, as knowledge of persuasion is particularly developing in these phases.

To start, cognitive abilities necessary for recognizing and processing sponsored online influencer content – such as logical reasoning and information processing – show a linear increase with age and stabilize by middle adolescence (Rozendaal et al. 2011). As a consequence, in early adolescence, knowledge of persuasion will not be easily triggered, making early adolescents more likely than adults to be influenced by advertising (Eagle 2007). Early adolescents’ cognition is characterized by concrete thinking (“here and now”), lacking an understanding of how a present action has a result in the future. Middle adolescents have some experience using complex thinking processes, and therefore their focus expands to include more philosophical and futuristic concerns. Moreover, a better understanding of the results of one’s actions has developed by middle adolescence. This means that middle adolescents’ knowledge of persuasion might be easier activated as their cognitive abilities to connect input (e.g., advertising) to an output (e.g., persuasion) is better developed.

While the cognitive part of the brain develops linearly with age and stabilizes by middle adolescence, the subcortical affective parts of the brain develop relatively faster and are hyperactive during early adolescence (Defoe et al. 2015). Hormonal changes are peaking during early adolescence, for girls from 10 to 12 years old and for boys around 12 to 14 years old (Valkenburg and Piotrowski 2017). This causes an imbalance between the cognitive and affective regions of the brain and can result in the hyperactive affective system overriding the cognitive system (Defoe et al. 2015). Early adolescents are, therefore, more biased toward arousing affective stimuli, which explains, for example, their increased engagement in risky behavior and susceptibility to peer influence (Albert et al. 2013). This imbalance makes the activation of knowledge of persuasion troublesome for adolescents in general, but early adolescents specifically, when exposed to sponsored influencer videos. Sponsored influencer videos often contain affective content, which limits adolescents’ cognitive control to stop and recognize the commercial message.

Besides the development of cognitive abilities during adolescence, this period is also marked by social-emotional development (Valkenburg and Piotrowski 2017) in which they form their identity. Not only their direct environment, such as peers or parents, can play a role in identity formation, but also media personas, such as TV characters, celebrities, and online influencers are crucial (Choukas-Bradley et al. 2015). In particular, early adolescents perceive online influencers as role models and seem to be extra susceptible to their opinion (Valkenburg and Piotrowski 2017). Adolescents are likely to mirror the attitudes and actions of their role models without critically examining whether this information is sincere or sponsored. Therefore, (early) adolescents are expected to activate their knowledge of persuasion less easily when exposed to sponsored influencers’ content.

### Effects of Conceptual Knowledge of Persuasion on Attitudinal Knowledge of Persuasion

Research has shown that the activation of people’s conceptual knowledge of persuasion can also activate attitudinal knowledge of persuasion (see for example, An et al. 2014; Boerman and van Reijmersdal 2016). Attitudinal knowledge of persuasion is defined as ‘critical attitudes toward advertising, for example, skepticism of disliking of the advertising’ (Rozendaal et al. 2011, p. 344). Activated conceptual knowledge of persuasion can increase the motivation to resist the message by promoting feelings of psychological reactance (Brehm and Brehm 1981). That is, being aware of sponsorship and/or its persuasive intent causes people to feel restricted in their freedom to think and feel what they want, which motivates them to actively restore this freedom by questioning and discounting advertising’s claims (Knowles and Linn 2004).

In a series of mediation tests, An et al. (2014) studied children’s (aged 8–9) knowledge of persuasion and showed that only those children who viewed the advergame as a type of advertising held more skeptical attitudes toward the advergame. This aligns with the findings of Boerman et al. (2017), who found in their study on sponsored Facebook posts that the activation of conceptual knowledge of persuasion caused higher levels of attitudinal knowledge of persuasion among adults. Similarly, De Jans et al. (2018) found that disclosing advertising in a sponsored video resulted in more conceptual and attitudinal knowledge of persuasion among adolescents age 11 to 14. When adolescents realize that the content is not just entertainment but has a persuasive intent, a change of meaning principle (Friestad and Wright 1994) might occur: when adolescents realize that the content they were watching is not meant to entertain them, but actually to sell them certain products, they can feel fooled and this realization might motivate them to process the content more critically. Hence, it is proposed that the recognition of sponsored content as being advertising and understanding persuasive intent (i.e., the activation of conceptual knowledge of persuasion) is followed by the activation of attitudinal knowledge of persuasion.

### Effects of Disclosures on Advertising Outcomes and Attitudes toward the Influencer

Disclosures that trigger conceptual (and consequently attitudinal) knowledge of persuasion serve as tools for adolescents to cope with the persuasive attempt (Friestad and Wright 1994). This could be by processing the content in a more critical way and choosing to either resist the persuasive attempt or to be persuaded. Attitudinal knowledge of persuasion has been shown to be effective in generating resistance among children (aged 5 to 11) and adults (e.g., Vanwesenbeeck et al. 2017). Critical feelings toward advertising have indeed been found to transfer to the advertised brand (MacKenzie et al. 1986).

Studies on the relationship between knowledge of persuasion and brand attitude and brand preferences among children and adolescents between 7 and 13 years old showed that activated knowledge of persuasion leads to a less favorable brand attitude and preferences within traditional advertising formats (Rozendaal et al. 2012). Regarding online embedded advertising, Rozendaal et al. (2013) found that attitudinal knowledge of persuasion was an effective defense among children aged 9 to 12, such that higher attitudinal knowledge of persuasion led to less favorable brand attitudes. However, for adolescents, Van Reijmersdal et al. (2017) showed that disclosures for brand placement in a television program partially activated adolescents’ (13 to 17 years old) conceptual knowledge of persuasion, that is, their understanding persuasive intent, but not their recognition of sponsored content as being advertising. Also, there was no significant effect found on brand attitude via attitudinal knowledge of persuasion.

Research on the effects of disclosures on purchase intention showed mixed findings: On the one hand, De Jans et al. (2018) found that disclosures for sponsored online videos resulted in lower purchase intentions among adolescents aged 11 to 14, but only through activated attitudinal knowledge of persuasion. Similarly, in their study on disclosures on television among children (8 to 12 years old), Rozendaal et al. (2016) found that disclosure of manipulative intent activated attitudinal knowledge of persuasion, which subsequently lowered product desire. This aligns with the study of An and Stern (2011) that showed a direct negative effect of a textual and graphic disclosure on children’s (8 to 11 years old) product desire. A study among adults showed similar results; a disclosure reduced movie watchers’ intention to purchase the advertised brand (Tessitore and Geuens 2013). On the other hand, Vanwesenbeeck et al. (2017) found a reversed effect in their study on advergames. They demonstrated that children of 10 to 12 years old with an increased understanding of the persuasive intent of the advergame were more likely to buy the advertised brand, which could be due to the specific and entertaining context of advergames.

In summary, it remains unclear whether the activation of knowledge of persuasion will mitigate advertising effects on adolescents in the context of sponsored online influencer content (e.g., purchase intention and brand attitude). In line with theoretical notions, such as the persuasion knowledge model and empirical evidence, it is expected that conceptual and attitudinal knowledge of persuasion, activated by a disclosure, can have a negative impact on adolescents’ brand attitude and purchase intention compared to content without a disclosure. However, disclosures might not only influence attitudes toward brands via activated knowledge of persuasion, but it can also change one’s view of the online influencer. In line with the PKM (Friestad and Wright 1994), it is expected that adolescents’ perceptions of an influencer will change when activating their conceptual knowledge of persuasion and realizing that the created content is not just for entertainment, but also serves commercial purposes. They can feel fooled by the influencer, potentially leading a less positive attitude toward the influencer. Additionally, the literature on embedded advertising in television programs found a spill-over effect (i.e., when attitudes toward one object influence the attitudes toward another) when knowledge of persuasion was activated among adults (Van Reijmersdal et al. 2010). Critical attitudes could spill over to the online influencer. Colliander and Erlandsson (2015) showed that a third-party disclosure of a sponsored blog results in a decreased perceived credibility of and attitude toward the blog among adults, compared to a sponsored blog without a disclosure. Noteably, Liljander et al. (2015) studied adults’ responses to brand recommendations in blogs but did not find any effects of either covert or overt marketing on influencer credibility. This study will examine whether disclosure types affect attitudes toward the influencer via conceptual and attitudinal knowledge of persuasion.

## Current Study

The components of conceptual knowledge of persuasion (i.e., recognition of sponsored content as being advertising and understanding the persuasive intent) are expected to be triggered more or less easily by specific information given in a disclosure (Van Reijmersdal 2016). A disclosure – providing information that the content contains advertising – is expected to trigger more knowledge of persuasion, especially recognition of sponsored content as being advertising, compared to no disclosure. Furthermore, a disclosure that additionally provides information about the advertising’s intentions is expected to increase not only recognition of advertising but also the second component, understanding of the persuasive intent of advertising. It is hypothesized that disclosure type affects recognition of sponsored content as being advertising, such that disclosure of advertising and disclosure of advertising and intent compared to no disclosure both lead to more recognition (Hypothesis 1a). In addition, it was expected that disclosure type affects understanding of persuasive intent, such that a disclosure of advertising and intent will lead to higher levels of understanding of persuasive intent compared to disclosure of advertising alone, which in turn will lead to higher levels of understanding persuasive intent compared to no disclosure (Hypothesis 1b).

Adolescents’ developmental stage is expected to play a moderating role in the effects of disclosure types on conceptual knowledge of persuasion. Taking into account cognitive and social-emotional developments, middle adolescents might need less information to activate higher levels of knowledge of persuasion compared to early adolescents. As they have more cognitive abilities and better-developed knowledge of persuasion, middle adolescents (compared to early adolescents) might already activate both components of conceptual knowledge of persuasion (i.e., recognition of sponsored content as being advertising and understanding of persuasive intent) by providing only disclosure of advertising.

Due to their limited cognitive and social-emotional development, early adolescents are expected to activate their recognition of sponsored content as being advertising in response to a disclosure of advertising, but they are expected to further enhance their recognition of advertising and to only active their understanding of the persuasive intent when exposed to a disclosure that gives explicit information on the advertising’s intent. More specifically, it is expected that age moderates the effect of disclosure type on recognition of sponsored content as being advertising, such that for a) middle adolescents both a disclosure of advertising and a disclosure of advertising and intent (compared to no disclosure) will lead to higher levels of recognition of sponsored content as being advertising and for b) early adolescents, a disclosure of advertising and intent will lead to the highest levels of recognition of sponsored content as being advertising, followed by a disclosure of advertising, followed by and no disclosure (Hypothesis 2).

With respect to the effect of disclosure type on understanding of advertising intent, it is expected that age plays a moderating role, such that a) for middle adolescents both a disclosure of advertising and a disclosure of advertising and intent (compared to no disclosure) will lead to higher levels of understanding persuasive intent, whereas b) for early adolescents, only the disclosure of advertising and intent (compared to no disclosure and disclosure of advertising) will lead to higher levels of understanding persuasive intent (Hypothesis 3).

Furthermore, the disclosures are expected to affect responses to the brand and the influencer via activated knowledge of persuasion: Disclosures make adolescents aware of the commercial intention (i.e., increased conceptual knowledge of persuasion) and subsequently lead to a more critical evaluation of the influencer (i.e., increased attitudinal knowledge of persuasion), which might spill over to adolescents’ evaluation of the brand and the influencer. In particular, it is hypothesized that a sponsorship disclosure will activate adolescents’ recognition of advertising and their understanding of persuasive intent, which both lead to higher attitudinal knowledge of persuasion that, results in less favorable attitudes toward the brand, lower purchase intentions, and less favorable attitudes toward the influencer (Hypothesis 4).

Combining the proposed moderation effects (hypothesis 2 and 3) with the proposed indirect effects (hypothesis 4), a moderated mediation effect is hypothesized, such that the effects of disclosure type on a) brand attitude, b) purchase intention, and c) influencer attitude (via recognition of advertising and attitudinal knowledge of persuasion) are moderated by age (Hypothesis 5). Also, the effects of disclosure type on a) brand attitude, b) purchase intention, and c) influencer attitude (via an understanding of persuasive intent and attitudinal knowledge of persuasion) are also expected to be moderated by age (Hypothesis 6).

## Method

### Design

To test the hypotheses, a 3 (disclosure: advertising vs. advertising and intent vs. no disclosure) × 2 (age: 12–14 vs. 15–16) experimental between-subject design was used. The original design also included a factor ‘explanation (absent or present)’. In the explanation condition, the participants were shown a text before watching the video that included an explanation of the possible relations between a brand and a YouTuber and how YouTubers can disclose this relationship. However, this explanation did not moderate the effects of disclosure types on conceptual knowledge of persuasion. Therefore, the explanation and no explanation groups were collapsed in the analyses reported here. The participants were randomly assigned to the conditions. The experiment took place at the adolescents’ schools and was conducted through an online survey tool (Qualtrics).

### Participants and Procedure

In total, 406 students from 12 to 16 years old from the Netherlands participated in the experiment. One student of 11 years old and five students of 17 years old were removed before the analyses because their age was outside the scope of this study. The average age was 14 (*SD* = 0.95), 56.6% were female. In the total sample, there were 167 early adolescents (aged 12–14 years, mean age 12.85, *SD* = 0.14, 53% female) and 239 middle adolescents (aged 15–16 years, mean age 14.36, *SD* = 0.13, 59% female). Adolescents were recruited from three schools in urban and suburban areas that provided different educational levels. Before participating, institutional approval, parental approval, and adolescent’s informed consent were obtained. The entire class, consisting of approximately 30 students, participated at the same time in the experiment using available computers, laptops, or tablets of their school. Participants were randomly assigned to one of the three disclosure conditions. Participants were asked to watch the video as they would normally do. Once they had watched the whole video, they could directly proceed with the questionnaire. The video and the questionnaire were in Dutch, since all participants were from the Netherlands. Control variables regarding the video (video familiarity, attitude toward video, influencer familiarity) and attitude toward the influencer were measured. Next, questions regarding the mediating variables (conceptual and attitudinal knowledge of persuasion) were asked, followed by the two other dependent variables, brand attitude and purchase intention. The questionnaire ended with a question on the manipulations (disclosure types) and additional control variables.

### Stimulus Materials

The current study focuses on influencer videos on YouTube for two reasons. First, a recent analysis of YouTube content showed that those who watch YouTube videos ‘are confronted with an ever-growing share of product promotions’ (Schwemmer and Ziewiecki 2018, p. 2). Second, recent reports show the immense popularity if watching YouTube videos among minors: YouTube content aimed at minors has been found to have higher view counts than any other kind of content on YouTube (Van Kessel et al. 2019). Also, online videos are more popular than most other forms of entertainment among children aged 8 and older (Common Sense 2019).

The stimulus material consisted of an existing YouTube video of a well-known Dutch YouTube influencer aged 25. He makes videos about his life and interests, which are sometimes sponsored or contain sponsored products. The video used for this study was sponsored by the Fanta soft drink brand. The influencer provides its viewers with ten tips to make life easier, whereby three of the ten tips involved Fanta. The product and the logo were clearly visible.

In the experimental disclosure conditions, during the first 10 s of the video, a disclosure was shown at the top of the screen. The disclosure of advertising consisted of the sentence: “[The influencer] is paid to promote Fanta during this video”, while the disclosure of advertising and intent was: “[The influencer] is paid to promote Fanta during this video, to make you like Fanta”. The participants in the control conditions were exposed to the video without any disclosure.

### Measures

Almost all items were measured on a 6-point scale ranging from 1 (*No, definitely not)* to 6 *(Yes, for sure)*. Children are known to have a tendency to choose a neutral mid-point when this is offered (Borgers et al. 2004). Therefore, a 6-point scale was used. Only video and influencer familiarity, attitude toward the video, and demographics were measured differently and will, therefore, be described separately.

#### Knowledge of persuasion

The elements of knowledge of persuasion were all measured with the validated scales of Rozendaal et al. (2016). These authors developed and tested these scales for minors. Recognition of sponsored content as advertising is measured with three questions: “Did this video contain advertising for a brand?” “Is this video sponsored by a brand?” and “Is this video a form of advertising?” Mean scores were calculated to create one recognition of advertising scale (Cronbach’s α *=* 0.81, *M* = 3.69, *SD=* 1.41). Understanding persuasive intent was measured with three questions: “Is the video made to make people buy Fanta?” “Is this video made to make people want Fanta?” and “Is this video made to make people like Fanta?” (Rozendaal et al. 2016). Mean scores were calculated to create one understanding of intent scale (Cronbach’s α *=* 91, *M* = 3.41, *SD=* 1.42). Attitudinal knowledge of persuasion is measured with four questions: “Do you think showing Fanta in the video is wrong/right (reversed), bad, honest (reversed)?” (Cronbach’s α = 0.72, M = 2.88, *SD* = 1.02; Rozendaal et al. 2016).

#### Brand attitude, purchase intention, and attitude toward the influencer

Brand attitude was measured with six questions: “Do you think Fanta is tasty, nice, bad (reversed), good, boring (reversed), stupid (reversed)?” (Rozendaal et al. 2012; Cronbach’s α *=* 0.79, *M* = 4.62, *SD* = 0.94). Purchase intention was measured with two questions: “Would you like to buy Fanta?” and “Are you going to buy Fanta?” (*r*_*SB*_= 0.68, *M* = 2.46, *SD* = 1.09). Attitude toward the influencer was measured with six questions: “Do you think [the influencer] is interesting, stupid (reversed), good, nice, bad (reversed), boring (reversed)?” (Batra and Stayman 1990; Cronbach’s α *=* 0.92, *M=* 4.24, *SD* = 1.30).

#### Control variables

There were several control variables included in this study to assure that the effects of the manipulation were not caused by other differences between the experimental groups than the manipulations. Video and influencer familiarity were measured by asking whether participants had already seen the video (0 = *No*, 1 = *Yes*), whether they knew the influencer (0 = *No*, 1 = *Yes*), and how often they watch videos of influencers with a response scale ranging from 1 (*Never*) to 6 (*Every day*).

Furthermore, attitude toward the video was measured by asking to rate the video on a scale of 1 to 10, ranging from negative to positive. Product category liking is measured by one item, asking whether the participants like soft drinks. The questions to measure product category use, advertised brand use, and medium use were all posed in the same way, namely how often the participants drink soft drinks, drink Fanta, and watch videos on YouTube. Responses ranged from 1 (*Never*) to 6 (*Every day*). Finally, the demographic variables grade, school level, sex, and age were measured.

To check whether the manipulation was successful, adolescents were asked for their disclosure recognition: “Did you see any text on screen?” with as answering options the advertising disclosure, the advertising and intent disclosure, “this is a YouTube video”, “[name influencer] created this video to earn money”, “[name influencer] likes to create videos”, and “I did not see a text”.

## Results

### Manipulation Checks

Confirmatory analyses regarding the success of the manipulation of disclosure type showed that there was a significant difference between the three conditions: control condition, advertising disclosure, and disclosure of advertising and persuasive intent, chi-squared (10) = 338.00, *p<* 0.001). Most of the adolescents in the control condition (79.7%) reported correctly that they had not seen a disclosure, 54.5% of the participants in the advertising disclosure condition recognized this disclosure, and 48.4% of the participants in the advertising and intent disclosure condition recognized the correct disclosure. In summary, those exposed to a disclosure reported a significantly higher recognition of the disclosure than those who did not see a disclosure, indicating a successful manipulation.

### Randomization Checks

Confirmatory analyses showed that the experimental conditions did not differ with respect to grade chi-squared (4) = 3.28, *p* = 0.51, school level chi-squared (4) = 2.00, *p* = 0.74, sex chi-squared (2) = 4.97, *p* = 0.08, influencer familiarity chi-squared (2) = 0.15, *p* = 0.93, video familiarity chi-squared (2) = 2.39, *p* = 0.30, how often they watch videos from the influencer *F* (2, 409) = 2.10, *p=* 0.12, attitude toward the video *F* (2, 409) = 3.07, *p=* 0.05, product category liking *F* (2, 409) = 1.04, *p=* 0.36, product category use *F* (2, 409) = 2.93, *p=* 0.06 advertised brand use *F* (2, 409) = 1.18, *p=* 0.31, or medium use *F* (2, 409) = 1.08, *p=* 0.34. This indicates successful randomization. However, the conditions did differ with respect to adolescents’ age category, chi^2^ (2) = 9.47, *p=* 0.01, but there was no multicollinearity (VIF = 1.001, Tolerance = 0.999), so age category could be used as a moderator of disclosure type effects in the analyses.

### Hypotheses Testing

To test Hypotheses 1 to 3, a MANOVA was conducted with disclosure type and age (12–13 vs. 14–16 years old) and their interaction as independent variables and recognition of sponsored content as advertising and understanding persuasive intent as dependent variables. With respect to Hypotheses 1a and 1b, the analysis showed a significant multivariate effect of disclosure type, Wilk’s Lambda = 0.916, *F*(4, 798) = 8.909, *p* < 0.001, partial eta^2 ^= 0.043. For both recognition of sponsored content as advertising, *F*(2, 400) = 13.555, *p* < 0.001, partial eta^2 ^= 0.063, and understanding the persuasive intent, *F*(2, 400) = 14.351, *p* < 0.001, partial eta^2 ^= 0.067, disclosure type had an effect. As expected, exposure to the disclosures of advertising and disclosure of advertising and intent led to a significantly higher recognition of sponsored content as being advertising than no disclosure (Table 1). There were no significant differences between the two disclosure types. As expected, disclosure of advertising led to a significantly higher understanding of persuasive intent than no disclosure, whereas disclosure of advertising and intent led to the highest level of understanding persuasive intent. Thus, H1 was supported.

**Table 1:** Effects of disclosure on recognition of sponsored content as being advertising and understanding persuasive intent

	Disclosure		
	Absent	Advertising	Advertising and intent
Recognition of advertising	3.200^a^ (1.388)	3.875^b^ (1.367)	4.131^b^ (1.281)
Understanding persuasive intent	2.973^a^ (1.387)	3.428^b^ (1.455)	3.948^c^ (1.218)

Mean scores are presented with standard deviations between parentheses

^a,b^Means with different superscripts in the same row differ significantly from each other using Bonferroni post hoc test with *p* < 0.05

**Fig. 1:**
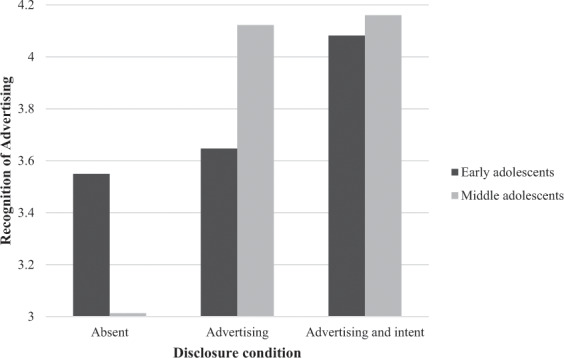
Interaction effects between disclosure type and age on recognition of sponsored content as being advertising

**Fig. 2:**
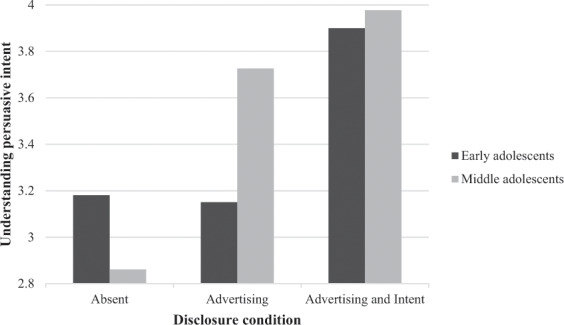
Interaction effects between disclosure type and age on understanding persuasive intent

With respect to Hypothesis 2 and 3, the analysis showed a significant multivariate interaction effect between disclosure type and age, Wilk’s Lambda = 0.974, *F*(4, 798) = 8.909, *p* = 0.032, partial eta^2 ^= 0.013. For both recognition of sponsored content as being advertising, *F*(2, 400) = 4.918, *p* = 0.008, partial eta^2 ^= 0.024, and understanding persuasive intent, *F*(2, 400) = 3.706, *p* = 0.25, partial eta^2 ^= 0.018, the interaction effect was significant. Post hoc analyses showed that for the early adolescents, the disclosure of advertising compared to no disclosure had no effect on recognition of sponsored content as advertising and understanding persuasive intent, but both recognition of sponsored content as advertising and understanding persuasive intent significantly increased with a disclosure of advertising and intent (Table 2 and Figs 1 and 2). For middle adolescents, a disclosure of advertising significantly increased their recognition of sponsored content as being advertising and understanding of persuasive intent, and this was not further enhanced by disclosure of advertising and intent. However, without a disclosure, recognition of sponsored content as being advertising was significantly higher for early adolescents than for middle adolescents, although both means were not above the mid-point of the scale. This indicates that both types of disclosures can significantly enhance recognition of sponsored content as being advertising and understanding persuasive intent among middle adolescents, but only disclosure of advertising and intent significantly enhances recognition of sponsored content as being advertising and understanding persuasive intent among early adolescents. This means that the data support H2 and H3.

**Table 2:** Means for recognition of sponsored content as being advertising and understanding intent

	Early adolescents			Middle adolescents		
	Disclosure			Disclosure		
	Absent	Advertising	Advertising and intent	Absent	Advertising	Advertising and intent
Recognition of advertising	3.549^a^ (1.467)	3.647^a^ (1.457)	4.082^b^ (1.341)	3.013^c^ (1.315)	4.122^b^ (1.227)	4.160^b^ (1.251)
Understanding persuasive intent	3.181^a^ (1.468)	3.151^a^ (1.436)	3.900^b^ (1.277)	2.861^a^ (1.336)	3.726^b^ (1.427)	3.977^b^ (1.188)

Mean scores are presented with standard deviations between parentheses

^a,b^Means with different superscripts in the same row differ significantly from each other using Bonferroni post hoc test with *p* < 0.05

With respect to H4, PROCESS macro (Hayes 2013; v3.0, Model 80, 10,000 bootstraps) was used with disclosure type (no disclosure, disclosure of advertising, disclosure of advertising and intent) as the categorical independent variable, recognition of sponsored content as advertising and understanding persuasive intent as mediators in parallel, and attitudinal knowledge of persuasion as a serial mediator, and either brand attitude, purchase intention, or influencer attitude as the dependent variable. This macro offers a test of mediation effects using a regression-based approach with bootstrapping. The analysis showed that there were no significant indirect effects of disclosure type on brand and influencer responses via conceptual knowledge of persuasion and, consequently, attitudinal knowledge of persuasion (Table 3). More specifically, the analyses showed that recognition of sponsored content as advertising and understanding persuasive intent were not significantly related to attitudinal knowledge of persuasion (*b* = 0.026, *SE* = 0.054, *t* = 0.491, *p* = 0.624; *b* = 0.102, *SE* = 0.054, *t* = 1.902, *p* = 0.058, respectively). Thus, higher conceptual knowledge of persuasion was not associated with higher attitudinal knowledge of persuasion. The analysis did show that higher attitudinal knowledge of persuasion led to more negative brand attitudes (*b* = −0.322, *SE* = 0.043, *t* = −7.406, *p* < 0.001) and more negative attitudes toward the influencer (*b* = −0.298, *SE* = 0.062*, t* = −4.784, *p* < 0.001) but did not affect purchase intent (*b* = 0.079, *SE* = 0.054. *t* = 1.458, *p* = 0.146). This means that more critical attitudes toward the sponsored content resulted in more negative attitudes toward the brand and the influencer, but left purchase intent unaffected. Thus Hypothesis 4 is rejected.

**Table 3:** Indirect effects of disclosure type on brand attitude, purchase intention and attitude toward the influencer via persuasion knowledge

Disclosure	Mediators	DV	*b*	SE	BCA95%CI
Absent vs. advertising	Recognition of advertising → apk	Brand attitude	−0.006	0.013	−0.032; 0.019
		Purchase intent	0.001	0.004	−0.006; 0.011
		Influencer attitude	−0.005	0.012	−0.030; 0.017
Absent vs. advertising and intent		Brand attitude	−0.007	0.016	−0.041; 0.025
		Purchase intent	0.002	0.005	−0.008; 0.014
		Influencer attitude	−0.007	0.015	−0.038; 0.024
Advertising vs. adverting and intent		Brand attitude	−0.002	0.005	−0.013; 0.007
		Purchase intent	0.000	0.002	−0.002; 0.004
		Influencer attitude	−0.002	0.005	−0.013; 0.007
Absent vs advertising	Understanding intent → apk	Brand attitude	−0.015	0.011	−0.040; 0.001
		Purchase intent	−0.004	0.004	−0.002; 0.015
		Influencer attitude	−0.014	0.011	−0.040; 0.000
Absent vs. advertising and intent		Brand attitude	−0.031	0.018	−0.071; 0.002
		Purchase intent	0.008	0.008	−0.003; 0.028
		Influencer attitude	−0.029	0.018	−0.070; 0.001
Advertising vs. adverting and intent		Brand attitude	−0.016	0.011	−0.042; 0.000
		Purchase intent	0.004	0.004	−0.002; 0.016
		Influencer attitude	−0.015	0.011	−0.041; 0.000

To test Hypotheses 5 and 6 on the moderated mediation effects, the PROCESS macro was used (Hayes 2013, v3.0, Model 83, 10.000 bootstraps). Disclosure type was treated as the categorical independent variable, the two age groups as moderators, and either recognition of sponsored content as being advertising or understanding persuasive intent as the first mediator, attitudinal knowledge of persuasion as the second mediator (serial), and either brand attitude, purchase intention, or attitude toward the influencer as dependent variable. The analyses showed that there were neither moderated mediation effects via recognition of sponsored content as advertising on brand attitude, purchase intention, or influencer attitude, nor via an understanding of persuasive intent when the effects of disclosure of advertising were compared to disclosure of advertising and intent (Table 4).

**Table 4:** Moderated mediation effect of disclosure type via recognition of sponsored content as advertising and attitudinal persuasion knowledge by age

Disclosure	Mediators	Dependent variable	Modmed index	*SE*	BCA95%CI
Absent vs. advertising	Recognition of advertising → apk	Brand attitude	**−0.033**	**0.018**	**−0.077; −0.006**
		Purchase intent	0.008	0.089	−0.003; 0.030
		Influencer attitude	**−0.031**	**0.018**	**−0.074; −0.005**
Absent vs. advertising and intent		Brand attitude	−0.020	0.015	−0.058; 0.002
		Purchase intent	0.005	0.006	−0.003; 0.022
		Influencer attitude	−0.019	0.015	−0.056; 0.002
Advertising vs. adverting and intent		Brand attitude	0.013	0.013	−0.009; 0.043
		Purchase intent	−0.003	0.004	−0.015; 0.004
		Influencer attitude	0.012	0.012	−0.009; 0.040
Absent vs. advertising	Understanding intent → apk	Brand attitude	−**0.034**	**0.019**	**−0.079; −0.005**
		Purchase intent	0.007	0.008	−0.004; 0.028
		Influencer attitude	**−0.031**	**0.019**	**−0.077; −0.004**
Absent vs. advertising and intent		Brand attitude	−0.015	0.015	−0.051; 0.009
		Purchase intent	0.003	0.005	−0.003; 0.017
		Influencer attitude	−0.014	0.015	−0.049; 0.009
Advertising vs. adverting and intent		Brand attitude	0.019	0.015	−0.006; 0.053
		Purchase intent	−0.004	0.005	−0.018; 0.003
		Influencer attitude	0.017	0.015	−0.006; 0.051

Numbers in bold font are significant at *p* < 0.05

*apk* attitudinal persuasion knowledge, *Modmed index* moderated mediation index, which is a unstandardized regression coefficients of the moderated mediation effect, *SE* standard error, *BCA95%CI* bias corrected accelerated 95% confidence interval

However, there were significant moderated mediation effects for the comparison between no disclosure and disclosure of advertising. The indirect effects of disclosing advertising (versus no disclosure) on brand attitude and influencer attitude via recognition of sponsored content as advertising and consequently attitudinal knowledge of persuasion was significant for middle adolescents (brand attitude: effect = 0.037, SE = 0.017, BC95%CI [−0.075; −0.010]; influencer attitude: effect = −0.034, SE = 0.017, BC95%CI [−0.072; −0.008]), but not for the early adolescents, (brand attitude: effect = 0.003, SE = 0.010, BC95%CI [−0.24; 0.025]; influencer attitude: effect = −0.003, SE = 0.009, BC95%CI [−0.023; 0.015]). Similarly, these effects on brand attitude and influencer attitude via understanding persuasive intent were significant for the middle adolescents (brand attitude: effect = −0.033, SE = 0.015, BC95%CI [−0.068; −0.009]; influencer attitude: effect = −0.030, SE = 0.016, BC95%CI [−0.068; −0.007]), but not for early adolescents (effect = −0.001, SE = 0.011, BC95%CI [−0.21; 0.023]; influencer attitude: effect = −0.001, SE = 0.010, BC95%CI [−0.020; 0.022]). This means that a disclosure of advertising (compared to no disclosure) significantly enhanced middle adolescents’ recognition of sponsored content as being advertising and their understanding of the persuasive intent of the sponsored video, which consequently enhanced their attitudinal knowledge of persuasion, ultimately resulting in less positive brand and influencer attitudes. But, the disclosure of advertising had no effects on brand attitudes and influencer attitudes among the early adolescents. There were no significant effects on purchase intention. Thus, H5a, H5c, H6a, and H6c are partially accepted, but H5b and H6b are rejected.

Additional exploratory analyses were performed to test whether the grade children were in or their school level played a moderating role in the effects of disclosure types on recognition of advertising or understanding intent. MANOVA showed that there were no multivariate direct or interaction effects of grade, Wilk’s Lambda = 0.986, *F*(4, 792) = 1.390, *p=* 0.236, eta^2 ^= 0.007 and Wilk’s Lambda = 0.993, *F*(8, 792) = 0.358, *p=* 0.942, eta^2^ = 0.004, respectively. There were also no multivariate direct or interaction effects of school level, Wilk’s Lambda = 0.979, *F*(4, 792) = 2.116, *p=* 0.077 and Wilk’s Lambda = 0.988, *F*(8, 792) = 0.596, *p=* 0.782, eta^2 ^= 0.011, respectively. These findings indicate that adolescents’ age phase moderates the effects of disclosure type on conceptual knowledge of persuasion, rather than the grade they are in or their school level.

## Discussion

To inform the audience that some posts by online influencers are sponsored by advertisers, disclosures are increasingly used. However, until now, little was known about how to formulate disclosures of influencer marketing to effectively inform adolescents about its persuasive nature. In addition, due to cognitive and social-emotional developmental differences between adolescents in early versus middle adolescents, it is expected that these groups of adolescents respond differently to disclosures. However, in previous studies on disclosures, adolescents’ developmental stages were not yet taken into account. Therefore, the present study aimed to enhance the understanding of how various disclosure types affect early and middle adolescents’ knowledge of persuasion and, consequently, how this affects brand and influencer responses In an experiment, two types of disclosures for sponsored influencer videos were tested (i.e., disclosing advertising, disclosing advertising and intent, or no disclosure) among early adolescents (12–14 years old) and among middle adolescents (15–16 years old). Based on the present study, at least two conclusions can be drawn.

First, this study shows that whether adolescents activate their knowledge of persuasion depends on both the content of the disclosure and on the adolescence phase they are in. Children in early adolescence need more information (on both advertising and intention) to activate the two components of conceptual advertising literacy (recognition of advertising and understanding persuasive intent) than children in middle adolescence. Just the disclosure of advertising activates middle adolescents’ understanding of influencers’ persuasive intention, whereas early adolescents need to be explicitly informed about the intention for the disclosure to activate that knowledge.

This reflects the predictions that were based on early adolescents’ cognitive and socio-emotional developments. First, previous research showed that early adolescents have less developed knowledge of persuasion (e.g., Lawlor and Prothero 2008; Verhellen et al. 2014), which seems to be confirmed by their lower levels of understanding persuasive intent when the disclosure is absent. It seems plausible that early adolescents learn from the disclosure of advertising intent and need this disclosure to understand the real intentions of the sponsored video. Second, middle adolescents are better able to connect an input to output (Eagle 2007), in this case, to relate the disclosure and the embedded advertising to persuasion. Third, the imbalance of cognitive and affective regions, which is more profound during early adolescents (Defoe et al. 2015), may make it harder for them to stop and think about the embedded advertising when limited information is provided in the disclosure.

Previous studies among children found no effects when disclosing brand placement in TV programs (van Reijmersdal et al. 2017), the selling intent of a commercial break (Rozendaal et al. 2016), or sponsorships within advergames (An and Stern 2011). Rozendaal et al. (2016) already started to explore information to include in the disclosure and showed that providing information on the TV commercial’s deceptive (versus promotional) nature was successful in increasing children’s advertising defenses through the activation of attitudinal knowledge of persuasion. The current study adds to this knowledge that adolescents’ age stage needs to be considered when deciding on the information that should be provided in a disclosure.

The second conclusion of the current study is that disclosure of sponsored influencer content does not decrease nor increase adolescents’ purchase intentions, via conceptual and attitudinal knowledge of persuasion. However, among middle adolescents, a disclosure of advertising (compared to no disclosure) activates recognition of sponsored content as being advertising and understanding of persuasive intent, which enhances attitudinal knowledge of persuasion and, consequently, results in a decrease in attitude toward the brand and toward the influencer. Thus, only middle adolescents might become more critical after seeing a disclosure, while this is not the case for early adolescents. This difference could be explained by the fact that early adolescence is characterized by concrete thinking and a lack of linking present actions to future results, while middle adolescents have an improved understanding of the consequences of their actions and are more experienced in using more complex thinking processes (Eagle 2007). Therefore, middle adolescents might be better able to link advertising to bias and misleading information (critical attitude) and see negative consequences for the brand and influencers as causers of this bias (attitude toward brand and influencer). Additionally, early adolescents are peaking in their sensitivity toward affective stimuli, such as influencer content (Albert et al. 2013), which limits their stop and think response, which is a mechanism that is necessary to use knowledge of persuasion as a defense and to trigger critical attitudes (Rozendaal et al. 2011). Overall, these findings show that middle adolescents show some resistance to persuasion by influencer videos due to disclosure, but that early adolescents do not show resistance (but also no persuasion) when compared to a situation without disclosure.

### Limitations and Suggestions for Future Research

This study has several limitations. First, the current study provides new insights regarding sponsored YouTube videos, while influencer marketing also finds its way onto social media outlets other than YouTube (e.g., Facebook, Instagram, Twitter). As posts on these platforms vary in format, and also these platforms might be used for various reasons (Voorveld et al. 2018), future studies should examine whether the effects of the disclosures used in the current study also hold for other social media platforms. On YouTube, there is space for spoken and written information, whereas Instagram, for example, has limited options for the influencer and is more visually focused, which might require adjusted disclosures.

This study focused on one specific video for a soft drink. The findings show that adolescents’ attitudes toward the brand were rather favorable, but their purchase intentions were rather low. This might indicate that adolescents are less susceptible to influencer marketing than may be expected based on their levels of knowledge of persuasion and their cognitive and social-emotional development. Future research is needed to examine whether the same effects hold for other types of products, for example, more expensive products or more high involvement products. Furthermore, the current study tested only the short-term effects of disclosures while empowering youth for the long term is desirable. It could be that adolescents forget this disclosure when watching, for example, another video of another influencer at a different time. As it is important to empower the youth for the long term, future research should focus on long-term and generalizing effects of disclosures on adolescents’ knowledge of persuasion. Additionally, future research should look further into the state and development of knowledge of persuasion of children within different age categories. The current study focused on early and middle adolescents, and the results show their different needs and requirements with regards to disclosure content. Future studies might compare this age group with either late adolescents or adults.

### Theoretical and Practical Implications

The findings of the current study have several theoretical and practical implications. First, the present study contributes to the understanding of information processing in adolescence. Adolescents show differential susceptibility to disclosure types across early and middle adolescence. The findings seem to imply that early adolescents learn from the disclosure that there is a persuasive intent and that this knowledge is not present in this age phase. The findings imply that early adolescents process entertaining and engaging information, such as sponsored influencer marketing videos, fundamentally differently than middle adolescents, which leads to lower levels of persuasion toward this type of advertising among early adolescents.

This study provides theoretical insights into adolescents’ knowledge of persuasion. Interestingly, this study does not conform to the assumption that conceptual knowledge of persuasion triggers a critical attitude toward the sponsored content for early adolescents (Rozendaal et al. 2016). For early adolescents and within this specific context, the activation of conceptual knowledge of persuasion due to a disclosure did not influence their attitudinal knowledge of persuasion. However, among middle adolescents, critical attitudes were activated by conceptual knowledge of persuasion that was activated by disclosure of advertising. As such, this study adds to the debate about the value of conceptual knowledge of persuasion as a critical defense against advertising. The use of knowledge of persuasion as a critical defense seems to reach a tipping point in middle adolescence. One’s age stage seems an important determinant of how children respond to disclosures and advertising, which emphasizes the urge for research on adolescents’ knowledge of persuasion and its development.

For policymakers, this study provides two important insights. First, the findings show what a disclosure should entail for adolescents within specific age stages in order to make them aware of influencer marketing and its intentions. While some authorities have already developed guidelines on how to disclose sponsored content (EASA 2018; Federal Trade Commission 2017), the effectiveness of disclosure types had not yet been empirically investigated among adolescents. The findings of this study indicate that not every disclosure is equally effective across adolescents’ age stages. As early adolescents need more information compared to middle adolescents to activate their conceptual knowledge of persuasion, it is recommended to create a disclosure that includes influencer marketing’s intentions to improve adolescents’ recognition and understanding of the sponsored online influencer content. When both advertising and the persuasive intent are disclosed early and middle adolescents will benefit.

For advertisers and influencers, this study has practical implications. Disclosures ensure a transparent media environment in an era where responsible marketing and ethical treatment of children is crucial. While disclosures do improve adolescents’ recognition and understanding of the sponsored message in influencer content, disclosures do not necessarily harm brand and influencer attitudes or buying intentions.

## Conclusion

Disclosures are used to inform audiences about the persuasive nature of influencer marketing. However, little was known about how to formulate these disclosures to help adolescents understand the commercial nature of this practice. In addition, knowledge on whether early adolescents respond differently to these disclosures than middle adolescents was missing. From the present study, we can conclude that early adolescents do not show an understanding of the persuasive intent of sponsored influencer videos when this information is not given in a disclosure. Middle adolescents can activate both recognition of advertising and their understanding of the persuasive intent of sponsored influencer videos when only the fact that the video contains advertising is disclosed. This indicates that middle adolescents have a better developed cognitive network concerning knowledge of persuasion. Middle adolescents can activate related concepts, such as understanding of advertising’s intent, when confronted with disclosure of advertising. Moreover, middle adolescents become more critical toward the brand and the influencer when advertising is disclosed, whereas early adolescents show no resistance. These findings add to the research on adolescence as they show that adolescents’ responses to influencer marketing are a function of their developmental stage in combination with disclosure content. More specifically, the fundamentally different ways of processing disclosures and sponsored influencer content that are found for early versus middle adolescents are likely to be attributed to early adolescents’ limited knowledge of persuasion (e.g., Lawlor et al. 2016; Verhellen et al. 2014), but also to their tendency to respond emotionally rather than cognitively (Defoe et al. 2015) and their social-emotional development, which makes them less likely to show resistance (Valkenburg and Piotrowksi 2017). The present study shows that not only disclosure content, but also the developmental phase of adolescence determines whether disclosures can effectively inform adolescents about the persuasive nature of influencer marketing.

## References

[CR1] Albert D, Chein J, Steinberg L (2013). The teenage brain: peer influences on adolescent decision making. Current Directions in Psychological Science.

[CR2] An S, Jin HS, Park EH (2014). Children’s advertising literacy for advergames: perception of the game as advertising. Journal of Advertising.

[CR3] An S, Stern S (2011). Mitigating the effects of advergames on children. Journal of Advertising.

[CR4] Batra R, Stayman DM (1990). The role of mood in advertising effectiveness. Journal of Consumer Research.

[CR5] Boerman SC, Willemsen LM, Van der Aa, Eva P (2017). “This post is sponsored:” effects of sponsorship disclosure on persuasion knowledge and electronic word of mouth in the context of Facebook. Journal of Interactive Marketing.

[CR6] Boerman SC, van Reijmersdal EA, De Pelsmacker P (2016). Informing consumers about “hidden” advertising: a literature review of the effects of disclosing sponsored content. Advertising in new formats and media: current research and implications for marketers.

[CR8] Borgers N, Hox J, Sikkel D (2004). Response effects in surveys on children and adolescents: the effect of number of response options, negative wording, and neutral mid-point. Quality and Quantity.

[CR9] Boush DM, Friestad M, Rose G (1994). Adolescent scepticism toward TV advertising and knowledge of advertiser tactics. Journal of Consumer Research.

[CR10] Brehm SS, Brehm JW (1981). Psychological reactance: a theory of freedom and control.

[CR11] Choukas-Bradley S, Giletta M, Cohen GL, Prinstein MJ (2015). Peer influence, peer status, and prosocial behavior: an experimental investigation of peer socialization of adolescents’ intentions to volunteer. Journal of Youth and Adolescence.

[CR12] Colliander J, Erlandsson S (2015). The blog and the bountiful: exploring the effects of disguised product placement on blogs that are revealed by a third party. Journal of Marketing Communications.

[CR13] Common Sense (2019). The common sense census: media use by tweens and teens 2019. https://www.commonsensemedia.org/research/the-common-sense-census-media-use-by-tweens-and-teens-2019?j=7514895&sfmc_sub=170952129&l=2048712_HTML&u=137031445&mid=6409703&jb=1412&utm_source=research_8-18census_2019&utm_medium=email.

[CR14] De Jans S, Cauberghe V, Hudders L (2018). How an advertising disclosure alerts young adolescents to sponsored vlogs: the moderating role of a peer-based advertising literacy intervention through an informational vlog. Journal of Advertising.

[CR15] De Veirman M, Cauberghe V, Hudders L (2017). Marketing through Instagram influencers: the impact of number of followers and product divergence on brand attitude. International Journal of Advertising.

[CR16] Defoe IN, Dubas JS, Figner B, van Aken MA (2015). A meta-analysis on age differences in risky decision making: adolescents versus children and adults. Psychological Bulletin.

[CR17] Eagle L (2007). Commercial media literacy: what does it do, to whom—and does it matter?. Journal of Advertising.

[CR18] EASA (2018). EASA best practice recommendation on influencer marketing. https://www.easa-alliance.org/news/easa/easa-launches-best-practice-recommendation-influencer-marketing-0.

[CR21] Federal Trade Commission (2017). The FTC’s revised endorsement guides: what people are asking. https://www.ftc.gov/tips-advice/business-center/guidance/ftcs-endorsement-guides-what-people-are-asking.

[CR22] Friestad M, Wright P (1994). The persuasion knowledge model: how people cope with persuasion attempts. Journal of Consumer Research.

[CR23] Gunter B, Oates C, Blades M (2005). Advertising to children on TV: content, impact and regulation.

[CR24] Hayes AF (2013). Introduction to mediation, moderation and conditional process analyses.

[CR25] Hudders L, De Pauw P, Cauberghe V, Panic K, Zarouali B, Rozendaal E (2017). Shedding new light on how advertising literacy can affect children’s processing of embedded advertising formats: a future research agenda. Journal of Advertising.

[CR26] John DR (1999). Consumer socialization of children: a retrospective look at twenty-five years of research. Journal of Consumer Research.

[CR27] Knowles ES, Linn JA, Knowles ES, Linn JA (2004). The importance of resistance to persuasion. Resistance and persuasion.

[CR28] Lapierre, M. A., & Rozendaal, E. (2019). A cross-national study examining the role of executive function and emotion regulation in the relationship between children’s television exposure and consumer behavior. *Journal of Youth and Adolescence*, *10*, 1–25. 10.1007/s10964-019-01119-7 31506774

[CR29] Lawlor MA, Dunne A, Rowley J (2016). Young consumers’ brand communications literacy in a social networking site context. European Journal of Marketing.

[CR30] Lawlor M, Prothero A (2008). Exploring children’s understanding of television advertising - beyond the advertiser’s perspective. European Journal of Marketing.

[CR31] Liljander V, Gummerus J, Söderlund M (2015). Young consumers’ responses to suspected covert and overt blog marketing. Internet Research.

[CR32] Livingstone S, Helsper EJ (2006). Does advertising literacy mediate the effects of advertising on children? a critical examination of two linked research literatures in relation to obesity and food choice. Journal of Communication.

[CR33] MacKenzie SB, Lutz RJ, Belch GE (1986). The role of attitude toward the ad as a mediator of advertising effectiveness: a test of competing explanations. Journal of marketing research.

[CR34] Pechmann C, Levine L, Loughlin S, Leslie F (2005). Impulsive and self-conscious: adolescents’ vulnerability to advertising and promotion. Journal of Public Policy & Marketing.

[CR35] Robertson TS, Rossiter JR (1974). Children and commercial persuasion: an attribution theory analysis. Journal of Consumer Research.

[CR36] Rozendaal, E., Buijs, L., & van Reijmersdal, E. A. (2016). Strengthening children’s advertising defenses: the effects of forewarning of commercial and manipulative intent. *Frontiers in Psychology*, *7*, 1186. 10.3389/fpsyg.2016.01186 PMC4976102 27551271

[CR37] Rozendaal E, Buijzen M, Valkenburg PM (2012). Think-aloud process superior to thought-listing in increasing children’s critical processing of advertising. Human Communication Research.

[CR38] Rozendaal E, Opree SJ, Buijzen M (2016). Development and validation of a survey instrument to measure children’s advertising literacy. Media Psychology.

[CR39] Rozendaal E, Buijzen M, Valkenburg PM (2010). Comparing children’s and adults’ cognitive advertising competences in the Netherlands. Journal of Children and Media.

[CR40] Rozendaal E, Lapierre MA, Van Reijmersdal EA, Buijzen M (2011). Reconsidering advertising literacy as a defense against advertising effects. Media Psychology.

[CR41] Rozendaal E, Slot N, Van Reijmersdal EA, Buijzen M (2013). Children’s responses to advertising in social games. Journal of Advertising.

[CR42] Schwemmer C, Ziewiecki S (2018). Social media sellout: the increasing role of product promotion on YouTube. Social Media+ Society.

[CR43] Spano S (2004). Stages of adolescent development: research FACTs and findings.

[CR45] Tessitore T, Geuens M (2013). PP for ‘product placement’ or ‘puzzled public’?. International Journal of Advertising.

[CR46] Valkenburg PM, Piotrowski JT (2017). Plugged in: how media attract and affect youth.

[CR47] Van Kessel, T., Toor, S. & Smith, A. (2019). A week in the life of popular YouTube channels. Pew Research Centre. https://www.pewresearch.org/internet/2019/07/25/a-week-in-the-life-of-popular-youtube-channels/.

[CR48] Van Reijmersdal EA, Boerman SC, Buijzen M, Rozendaal E (2017). This is advertising! effects of disclosing television brand placement on adolescents. Journal of Youth and Adolescence.

[CR49] Van Reijmersdal EA (2016). Disclosing brand placements in movies: effects of disclosure type and movie involvement on attitudes. Journal of Media Psychology: Theories, Methods, & Applications.

[CR50] Van Reijmersdal EA, Smit EG, Neijens PC (2010). How media factors affect audience responses to brand placement. International Journal of Advertising.

[CR51] Van Reijmersdal EA, Tutaj K, Boerman SC (2013). The effects of brand placement disclosures on skepticism and brand memory. Communications: The European Journal of Communication Research.

[CR52] Vanwesenbeeck, I., Opree, S. J., & Smits, T. (2017). Can disclosures aid children’s recognition of TV and website advertising? In V. Zabkar & M. Eisend (Eds), *Advances in advertising research VIII* (pp. 45–57). Wiesbaden: Springer Gabler.

[CR53] Verhellen Y, Oates C, De Pelsmacker P, Dens N (2014). Children’s responses to traditional versus hybrid advertising formats: the moderating role of persuasion knowledge. Journal of Consumer Policy.

[CR54] Voorveld HA, van Noort G, Muntinga DG, Bronner F (2018). Engagement with social media and social media advertising: the differentiating role of platform type. Journal of Advertising.

[CR55] Wojdynski BW, Evans NJ (2016). Going native: effects of disclosure position and language on the recognition and evaluation of online native advertising. Journal of Advertising.

[CR56] Wojdynski, B. W., & Evans, N. J. (2019). The covert advertising recognition and effects (CARE) model: processes of persuasion in native advertising and other masked formats. *International Journal of Advertising*. Advance online publication.

